# Plant-aphid interactions: recent trends in plant resistance to aphids

**DOI:** 10.1007/s44154-025-00214-z

**Published:** 2025-04-29

**Authors:** Kifle Gebreegziabiher Gebretsadik, Zhixin Liu, Jincheng Yang, Hao Liu, Aizhi Qin, Yaping Zhou, Enzhi Guo, Xiao Song, Peibo Gao, Yajie Xie, Ninkuu Vincent, Lam-Son Phan Tran, Xuwu Sun

**Affiliations:** 1 State Key Laboratory of Cotton Biology, Key Laboratory of Plant Stress Biology, School of Life Sciences, Henan University, 85 Minglun Street 85 Minglun Street, Kaifeng, 475001 People’s Republic of China; 2 Department of Plant and Soil Science, Institute of Genomics for Crop Abiotic Stress Tolerance, Texas Tech University, Lubbock, TX 79409 USA; 3 Tigray Agricultural Research Institute (TARI), Mekelle, 5637 Ethiopia

**Keywords:** Economic damage, Host plant resistance, Defense mechanisms, Physical defense, Secondary metabolites, Phytohormone, Green leaf volatiles

## Abstract

Aphids are highly destructive agricultural pests characterized by complex life cycles and phenotypic variability, facilitating their adaptation to diverse climates and host plants. Their feeding behavior leads to plant deformation, wilting, stunted growth, disease transmission, and significant yield losses. Given the economic risks aphids pose, regular updates on their seasonal behaviors, adaptive mechanisms, and destructive activities are critical for improving management strategies to mitigate crop losses. This review comprehensively synthesizes recent studies on aphids as plant pests, the extrinsic factors influencing their life cycles, and the intricate interactions between aphids and their hosts. It also highlights recent advancements in biological control measures, including natural enemies, antibiosis, and antixenosis. Additionally, we explore plant defense mechanisms against aphids, focusing on the roles of cell wall components such as lignin, pectin and callose deposition and the genetic regulations underlying these defenses. Aphids, however, can evolve specialized strategies to overcome general plant defenses, prompting the development of targeted mechanisms in plants, such as the use of resistance (R) genes against specific aphid species. Additionally, plant pattern recognition receptors (PRRs) recognize compounds in aphid saliva, which triggers enhanced phloem sealing and more focused immune responses. This work enhances understanding of aphid–plant interaction and plant resistance and identifies key research gaps for future studies.

## Introduction

Aphids, members of the order *Hemiptera*, superfamily *Aphidoidea*, and family *Aphididae*, are among the world’s most significant agricultural pests (Mou et al. 2023). Their rapid life cycle, diverse reproductive strategies, and broad environmental adaptability exacerbate their destructive impact on crop production (Dampc et al. 2020) (Dampc et al. 2020; Adhikari et al. 2019). The economically most impactful aphids are predominantly from the subfamily *Aphidinae*, comprising numerous species that infest herbaceous plants (Lin et al. 2022). Some of the economically damaging species include *Aphis gossypii* (Ramalho et al. 2012), *Myzus persicae* (Yang et al. 2023; Ahmed et al. 2022), *Diuraphis noxia* (Nicholson et al. 2015), *Sitobion miscanthi* (Zhang et al. 2022b; Wang et al. 2016), and *Rhopalosiphum padi* (Singh et al. 2020) (Table 1). Most aphids consume a limited number of host plants from a single family, while less than 1% can feed on plants from diverse families (Shih et al. 2023). Genetic variation in aphid populations, often associated with specific host plants, produces specialized biotypes adapted to a narrow range of hosts (Shih et al. 2023). Additionally, natural selection and migration contribute to the genetic diversity observed within aphid populations (Liu et al. 2020).

**Table 1:** Economically important aphid species and their host plants

Aphid species	Host plants	Reference
*Aphis gossypii*	*Gossypium hirsutum*, *Cucumis sativus*, *Capsicum annuum*, *Solanum tuberosum*, *Nicotiana tabacum*	(Ramalho et al. 2012)
*Acyrthosiphon gossypii*	*Gossypium hirsutum*, and legumes	(Gao et al. 2013)
*Myzus persicae*	Brassicaceae, Solanaceae, Cucurbitaceae, Rosaceae family	(Yang et al. 2023; Ahmed et al. 2022)
*Diuraphis noxia*	Poaceae family (*Triticum Aestivum*, *Hordeum vulgare*)	(Nicholson et al. 2015)
*Rhopalosiphum maidis*	Poaceae family (*Sorghum bicolor, Zea mays*)	(Maanju et al. 2023)
*Rhopalosiphum padi*	Poaceae family (*Triticum Aestivum*)	(Singh and Joshi 2020)
*Rhopalosiphum rufiabdominalis*	*Triticum Aestivum*, *Sorghum bicolor*, *Hordeum vulgare*	(Gill and Kunkel 2021)
*Sitobion miscanthi*	Poaceae family (*Triticum Aestivum*)	(Zhang et al. 2022b; Wang et al. 2016)
*Aphis glycines*	*Glycine max*, *Solanum tuberosum*, *brassica rapa*	(Bhusal et al. 2021)
*Aphis fabae*	*Vicia faba*	(Skovgård and Stoddard 2023)
*Aphis craccivora*	*Medicago sativa, Robinia pseudoacacia*	(Brady and White 2013)
*Therioaphis maculata*	*Medicago sativa*	(Dillwith et al. 1991)
*Schizaphis graminum*	Over 70 graminaceous species, including *Triticum Aestivum, Hordeum vulgare, Sorghum bicolor, Zea mays*	(Royer et al. 2015; Anstead et al. 2003)
*Melanaphis sacchari*	*Sorghum bicolor*	(Bowling et al. 2016)
*Acyrthosiphon pisum*	*Vicia fabae, Pisum sativum, Medicago sativa, Trifolium pratense,*	(Lv et al. 2023; Rahman et al. 2023)

Aphids possess specialized mouthparts, known as stylets, which enable them to extract phloem sap from plant tissues such as leaves, fruits, flowers, and stems (Shih et al. 2023). Their feeding behavior damages young shoots, causing twisting, stunting, curling, and withering of plant parts (Silva-Sanzana et al. 2020; Jasrotia et al. 2022). In addition to direct damage, aphids are vectors of plant diseases, spreading pathogens through localized feeding and long-distance migration (Gadhave et al. 2020). Their feeding activity also leads to honeydew deposition, which reduces photosynthetic efficiency, promotes sooty mold growth, and accelerates leaf aging (Jasrotia et al. 2022; Kutty and Mishra 2023).

Environmental factors, including topography and abiotic conditions, significantly shape aphid population dynamics, especially under changing climate conditions. For instance, temperature and altitude affect individual-level parameters, such as nymphal development time, adult longevity, and fecundity, as well as population-level parameters, including reproductive rate, intrinsic and finite rates of increase, and the generation time of *S. miscanthi* (Sun et al. 2022). Warmer climates typically increase aphid reproduction, but extremely high temperatures reduce survival. Optimal conditions for *S. miscanthi* growth range from 15–25 °C with 50–80% relative humidity (Sun et al. 2022). However, minor increases in temperature beyond this range adversely affect aphid development, metabolism, and reproduction, potentially intensifying crop damage (Adhikari et al. 2022; Deutsch et al. 2018). Notably, natural predators of aphids are less effective in areas with reduced biodiversity and increased agricultural intensification (Adhikari et al. 2019). Understanding the interactions between climate, landscape, and other environmental factors is essential for mitigating aphid-driven crop losses.

**Fig. 1:**
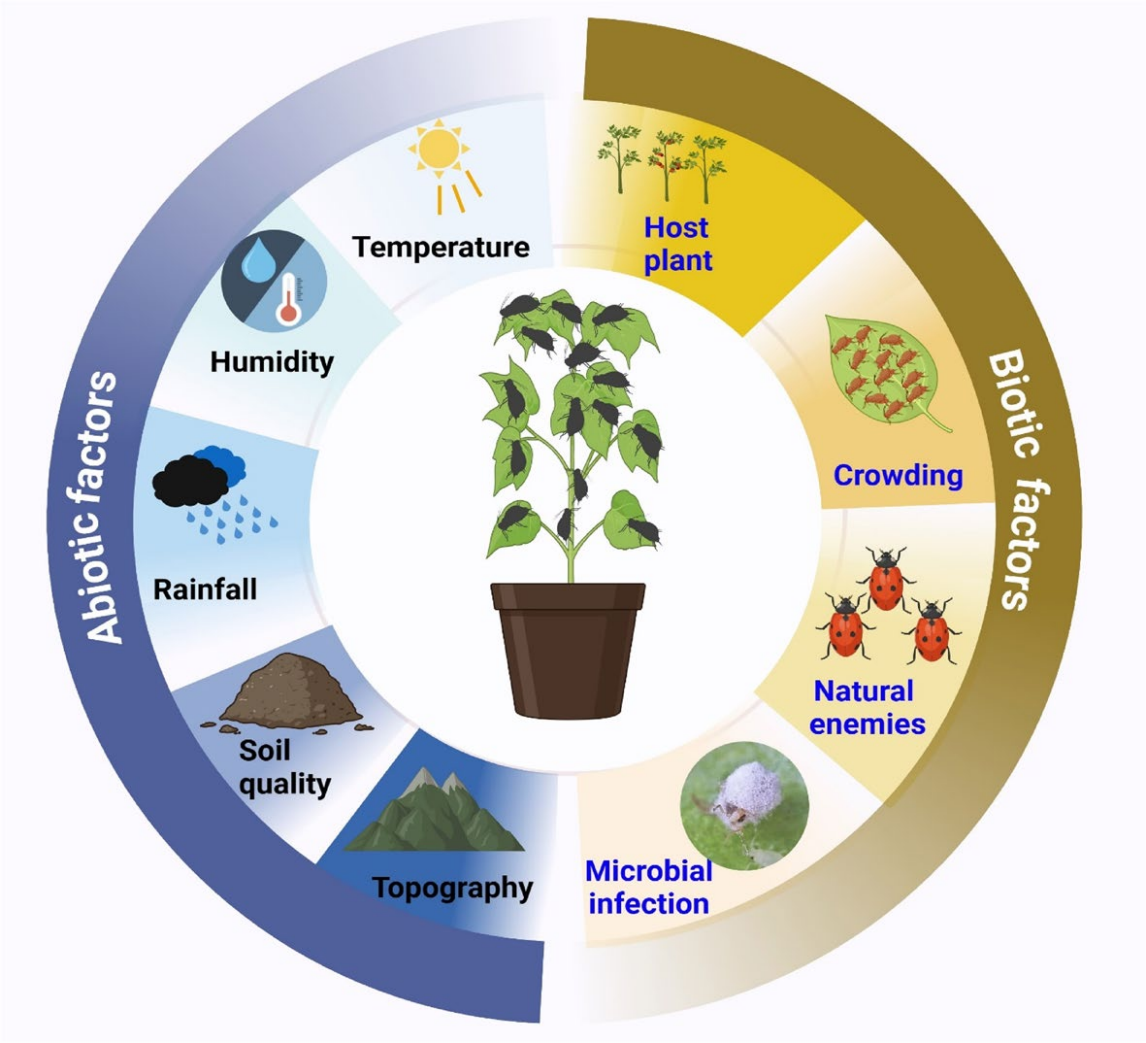
Extrinsic factors affecting aphid populations. Extrinsic factors, including abiotic factors (such as temperature, humidity, rainfall, soil quality, and topography) and biotic factors (such as host plants, crowding, natural enemies, and microbial infections), can significantly influence aphid populations

**Fig. 2:**
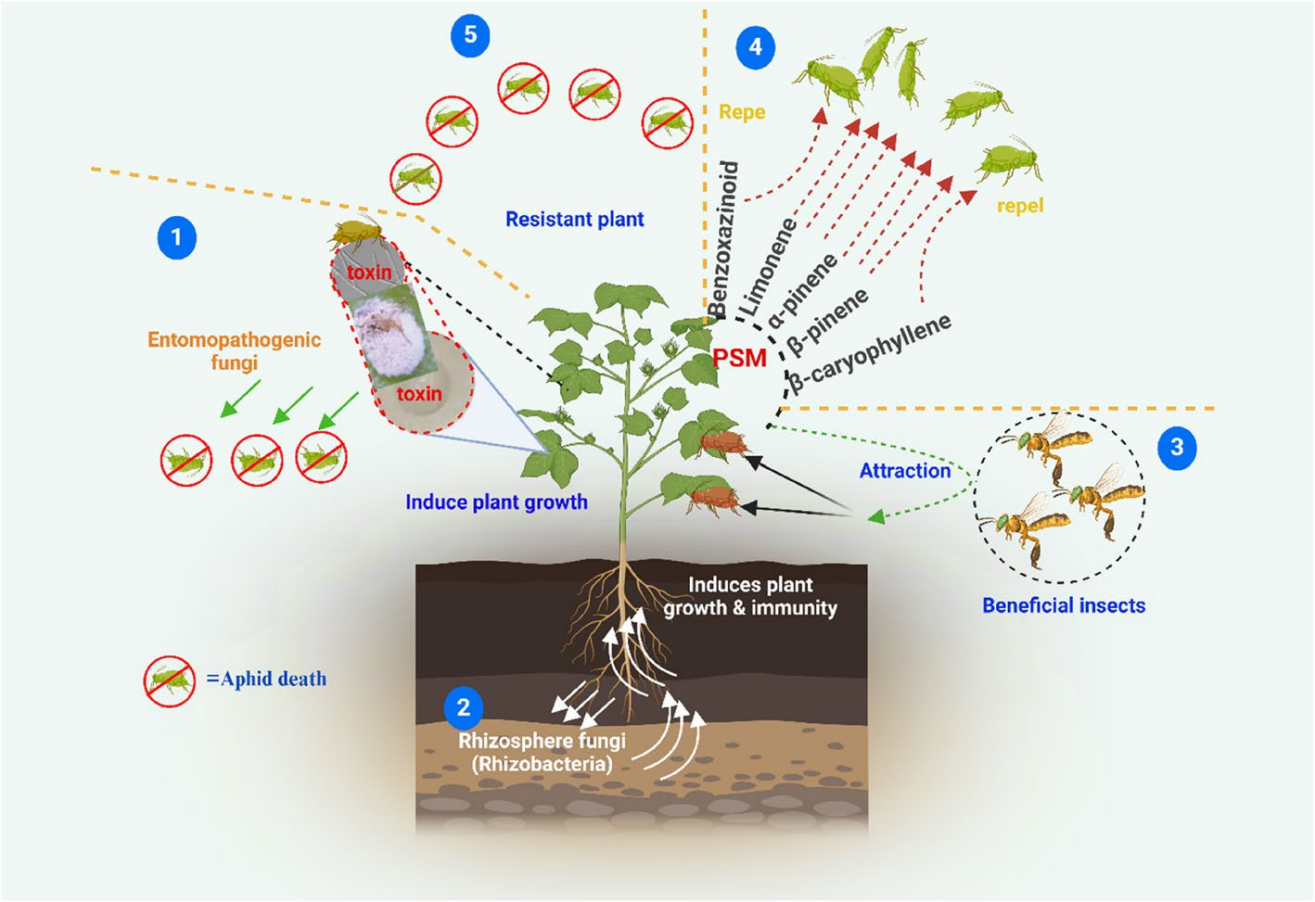
Induced systemic resistance mechanisms in plants against aphids. (1) Green arrows indicate aphid mortality caused by entomopathogenic fungi and toxins produced by these fungi. (2) White arrows represent rhizobacteria that enhance soil fertility, promote plant health, and confer resistance to aphids. (3) Black arrows depict the attraction of natural enemies by plant volatiles released from plants following an aphid attack. (4) Scattered red lines represent PSMs, such as benzoxazinoids, limonene, α-pinene, and β-caryophyllene, that deter aphids. (5) Resistant plants produce antifeedant and/or toxic substances that result in aphid mortality

Biotic factors, such as crowding, host plant quality, natural enemies (e.g., ladybugs, lacewings, wasps), and microbial infections, influence aphid survival and behavior (Sana et al. 2022). Entomopathogenic fungi (EPFs), including *Beauveria bassiana*, *Metarhizium anisopliae*, *Lecanicillium lecanii*, and others, effectively infect and kill aphids (Singh and Joshi 2020; Fingu-Mabola et al. 2021; Abdelaziz et al. 2018; Sun et al. 2023; Bayındır Erol et al. 2020; Saruhan 2018) (Table 2). EPFs also stimulate plant defense compounds, such as benzoxazinoids, terpenes, and flavonoids, which deter aphids (Lacey et al. 2015; Francis et al. 2022; Gange et al. 2019) (Fig. 1). Furthermore, plant growth-promoting rhizobacteria (PGPR), including *Bacillus* and *Pseudomonas* species, enhance plant health by inducing systemic resistance (ISR), activating immunity and antimicrobial responses. These responses inhibit aphid growth and increase their vulnerability to predators. The use of specific PGPR strains, such as *Bacillus* and *Pseudomonas*, offers promise in integrated pest management strategies (Finkel et al. 2017; Francis et al. 2022) (Fig. 2). Aphids maintain a close association with endosymbionts and serve as primary vectors of plant pathogens, particularly viruses (Zytynska et al. 2021; Eigenbrode et al. 2018; Mauck et al. 2018). These viruses significantly alter plant traits, including defense mechanisms, nutritional quality, and volatile emissions (Llave 2016; Pan et al. 2021; Mauck et al. 2014). Such changes influence aphid recruitment to and dispersal from infected plants, as well as the likelihood of virus acquisition during feeding (Ingwell et al. 2012; Pan et al. 2021; Fereres and Moreno 2009). These dynamics can, in turn, impact aphid population growth and the rate of virus transmission to new hosts (Shaw et al. 2017; Bak et al. 2017; Wu et al. 2014). Moreover, once aphid vectors acquire viruses, the pathogens can directly modify aphid traits, including locomotion and preferences for olfactory and visual cues related to infected and uninfected host plants (Wang et al. 2020; Chesnais et al. 2020).

**Table 2:** Entomopathogenic fungus and its target aphid species

Fungus species	Application method	Aphid species	References
*Beauveria bassiana, Metarhizium acridum*	2 ml of 10^8^ spores/ml suspension sprayed	*Myzus persicae, Rhopalosiphum padi*	(Fingu-Mabola et al. 2021)
*Beauveria bassiana, Cladosporium cladosporioides, and Verticillium alfalfae*	Aphids dipped in 10^7^ conidia ml^−1^ suspension for 10 s	*Metopolophium dirhodum*	(Abdelaziz et al. 2018)
*Beauveria bassiana*	Aphids were placed to crawl on conidia plates for 30 min	*Brevicoryne brassicae*	(Sun et al. 2023)
*Lecanicillium lecanii, Beauveria bassiana, Metarhizium anisopliae*	Foliar applications	*M. persicae*	(Singh and Joshi 2020)
*Lecanicillium muscarium, Simplicillium lamellicola*	2 ml of a solution containing 1 × 10^6^ conidia ml^−1^ was sprayed onto 10 aphids in Petri dishes using a Potter spray tower	*Aphis fabae*	(Saruhan 2018)
*Beauveria bassiana, Verticillium alfalfae, Trichoderma viride*	10^7^ conidia/ml solutions were applied using a hand sprayer	*Aphis gossypii*	(Bayındır Erol et al. 2020)
*Verticillium lecanii, Beauveria bassiana*	The leaf is dipped in a conidial solution and placed in a Petri dish with 50 nymphs	*Megoura japonica*	(Trinh et al. 2020)
*Metarhizium anisopliae, Metarhizium acridum*	Foliar applications	*Myzus persicae*	(Shan and Feng 2010)
*Beauveria bassiana SD15, Metarhizium anisopliae SD3*	An SD tower sprayer sprayed 1 ml of 108 conidia ml-1 suspension into a Petri dish	*Myzus persicae*	(Yun et al. 2017)

Aphid control strategies include cultural practices, chemical pesticide application, biological control methods, and recent advancements in utilizing host-plant resistance mechanisms to develop aphid-resistant cultivars (Chen 2023). Plants have evolved various genetic defense strategies against aphids, such as antibiosis, antixenosis, and tolerance (Painter 1951). In turn, aphids have developed specialized adaptations to overcome general plant defenses (Zust and Agrawal 2016; Nalam et al. 2019). To counter these adaptations, plants have established more targeted defense systems, primarily through resistance (R) genes and their homologs, which provide resistance against specific aphid species (Smith 2006). Additionally, plant pattern recognition receptors (PRRs) detect specific compounds in aphid saliva, triggering phloem sealing and other targeted immune responses (Chaudhary et al. 2014; Yang et al. 2024). While PRR- and R gene-mediated recognition pathways are distinct, both enable plants to recognize aphid feeding and initiate precise immune defenses. Despite the environmental and biodiversity risks posed by chemical control, it remains the dominant method due to its rapid efficacy in suppressing aphid populations (Luo et al. 2022; Mukherjee and Ghosh 2022). However, there is an urgent need to develop integrated, multifaceted approaches by comprehensively studying aphid biology and evaluating alternative control strategies to minimize reliance on chemical pesticides. This review provides a detailed analysis of recent literature on aphids as destructive agricultural pests, their economic impact, feeding behaviors, and external factors influencing their activity. Additionally, it highlights host resistance mechanisms mediated by cell wall polymers, which serve as physical and chemical barriers to aphid infestation. Overall, this review synthesizes current findings on aphid-induced economic losses, management strategies, and critical research gaps to guide future investigations.

### Aphid-plant interaction: the showdown of supremacy

Aphids are closely associated with their host plants, which provide both food and habitat, thereby influencing their evolutionary trajectories (Peccoud et al. 2010). Selective pressures exerted by plant chemical defenses drive the coevolutionary dynamics between aphids and plants, resulting in specific adaptations in certain aphid species for their preferred hosts (Becerra 1997; Peccoud et al. 2010). Some aphids utilize behavioral and chemical strategies to identify their host plants, influencing their specialization (Dixon 1998). However, the ability of some species to shift to different plants suggests that host specificity extends beyond mere resource adaptation (Peccoud et al. 2010). Additionally, host plants provide essential nutrients and mating sites (Dixon 1998), suggesting that constraints on host range are closely tied to mating success.

Aphids are small, sap-feeding insects that infest a variety of plants, which serve as their host plants. Over the course of evolution, aphids and their host plants have developed intricate relationships, with each influencing the other's traits and adaptations (Wooley et al. 2020). Aphids have evolved various mechanisms to overcome plant defenses, including the production of detoxifying enzymes and the adoption of diverse feeding strategies (Nalam et al. 2019). In response, host plants have developed various defenses, including toxic compounds and physical barriers (Mithofer and Maffei 2017). This ongoing "arms race" has driven the coevolution of specialized adaptations, influencing the host preferences and feeding behaviors of different aphid species.

**Fig. 3:**
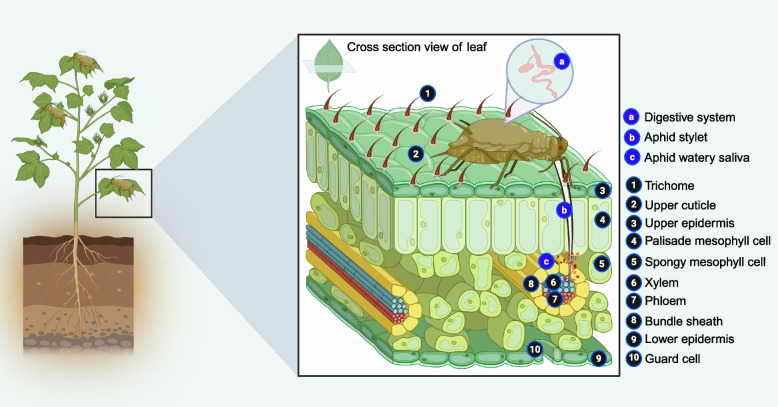
Graphic illustration of the aphid-plant interaction system. (1) Trichomes; (2) The upper cuticle, which serves as a physical barrier or chemical deterrent to aphids; (3) The upper epidermis; (4) The palisade mesophyll cells, which contain dense cells and thicker cell walls, providing additional physical barriers or chemical deterrents to protect plants from aphid stylet penetration; (5) The spongy mesophyll cells; (6) The xylem; (7) The phloem; (8) The bundle sheath; (9) The lower epidermis; (10) The guard cells. (a) Digestive system; (b) Aphid stylet; (c) Watery saliva, which is secreted by aphids into sieve tubes when ingesting phloem sap. This saliva helps protect capillary food canals from clogging with phloem proteins and detoxifies defensive substances in the phloem. Aphids use their stylets to penetrate plant tissues, which are adapted for piercing cell walls and accessing the nutrient-rich phloem sap. Aphids inject saliva containing a complex mixture of proteins that suppress plant immune defenses. In turn, plants detect these salivary secretions via molecular patterns, triggering defense signaling pathways. Upon detection of aphid presence, plants produce toxic compounds or proteins that directly harm or inhibit aphids. Additionally, plants may develop thicker cell walls to impede aphid stylet penetration and release volatile VOCs that attract natural enemies

Phloem sap is nutritionally rich and is a vital reservoir for various sap-sucking insects (Jiang et al. 2019). Phloem tissues comprise diverse and interactive cell types, which must be considered when analyzing the relationship between plants and phloem-feeding insects (Jiang et al. 2019). Aphids use their stylets to penetrate plant cell layers and access the phloem for essential nutrients, supporting their survival and development (Ahmed et al. 2022; Wang et al. 2021a) (Fig. 3). During probing and feeding, aphids secrete gelling and watery saliva into plant cells (Zhang et al. 2022a; Jiang et al. 2019) (Fig. 3). Gelling saliva forms a continuous salivary sheath along the stylet, which strengthens, lubricates, and protects against plant defense compounds (Huang et al. 2023; Will and Vilcinskas 2015) (Fig. 3). In contrast, watery saliva contains a complex mixture of bioactive molecules, including proteins, enzymes, carbohydrates, phospholipids, and other compounds, which modulate plant physiology and immune responses (Deshoux et al. 2022; Gao et al. 2023) (Fig. 3). Many components of watery saliva, such as Ca^2+^-binding proteins, proteolytic enzymes, and oxidizing effectors, play critical roles in aphid-plant interactions (Ahman et al. 2019). For instance, several salivary proteins from *Myzus persicae* (e.g., MP1, MP2, MP55, and MPMIIF1) inhibit host plant defense mechanisms, facilitating successful feeding and reproduction (Wang et al. 2021b). However, other salivary proteins can induce plant defense responses by promoting the production of toxic compounds, reinforcing cell walls, or releasing attractants that signal natural enemies (Guo et al. 2020). Interestingly, proteins such as MP10, MP42, MP56, MP57, and MP58 reduce the virulence of *M. persicae* (Wang et al. 2021b). In response, plants have evolved sophisticated mechanisms to counteract aphid effectors, ensuring a dynamic co-evolutionary arms race between plants and aphids.

Plant defenses can hinder aphid feeding and disrupt their nutrient acquisition from host plants (Shih et al. 2023; Xia et al. 2023). The deep-seated location of phloem sap within plants further limits aphid access (Twayana et al. 2022) (Fig. 3). One of the key plant defenses against aphids is the formation of sieve tube blockages (Kloth and Kormelink 2020). Plants have evolved both short- and long-term mechanisms to prevent phloem sap loss following aphid damage (Peng and Walker 2020). Short-term occlusion involves the rapid activation of phloem protein forisomes, particularly in legumes, in response to aphid feeding (Paulmann et al. 2023). These forisomes undergo reversible expansion, fully clogging the sieve elements upon Ca^2+^ influx triggered by aphid activity (Peng and Walker 2020). In contrast, callose deposition also mediates long-term occlusion induced by Ca^2+^ signaling (Hafke et al. 2009; Paulmann et al. 2023). Callose effectively blocks sieve tubes, preventing aphids from accessing nutrients (Kim et al. 2020; Guo et al. 2020). Additionally, plants generate reactive oxygen species (ROS) as a defense mechanism against aphids (Goggin and Fischer 2021; Nalam et al. 2019). ROS are highly reactive molecules capable of damaging cells, including those at the aphid-feeding site (Shih et al. 2023). These multi-layered defense strategies highlight plants' complex and dynamic responses to deter aphid infestation.

### Types of plant resistance to aphids

**Fig. 4:**
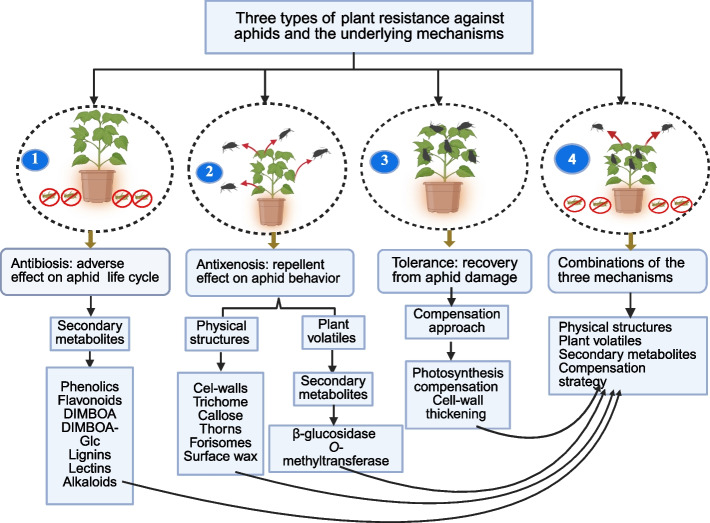
Three types of plant resistance against aphids and the underlying mechanisms.(1) Antibiosis; (2) Antixenosis; (3) Tolerance; (4) Combinations of the Three Mechanisms

Plants exhibit resilience against damage inflicted by insect pests (Subedi et al. 2023). Herbivores adapt to plant defense traits that have evolved in response to the consumption of plant tissues (Perkovich and Ward 2022). Plant defenses have evolved as a result of interactions with herbivores (Agrawal 2020; Perkovich and Ward 2022). These defenses include the synthesis of plant-derived compounds, optimization of foliar nutritional value, and redistribution of nutrients within the plant (Son et al. 2022; Perkovich and Ward 2022). Plants resist insect herbivores through antibiosis, antixenosis, tolerance, or combinations of these mechanisms (Koch et al. 2016; Leybourne and Aradottir 2022; Painter 1951) (Fig. 4). Antibiosis and antixenosis resistance arise as direct responses to plant–herbivore interactions, while tolerance represents the plant's broader, compensatory response to herbivory (Gebretsadik et al. 2022a; Peterson et al. 2017). Notably, a single host plant can exhibit multiple resistance mechanisms simultaneously (Radchenko et al. 2022). Studies have shown that these resistance traits are regulated at the genomic level. For instance, in sorghum (*Sorghum bicolor*), *Staygreen1* (*SGR1*), *Shoot gravitropism 2* (*SGR2*), *Shoot Gravitropism 7* (*Sgr7*), and *Shoot gravitropism* (*SGR8*) contribute to antibiosis and antixenosis resistance against *S. graminum.* Additionally, wheat accessions carrying the genes *Gb3*, *Gbx*, and *Gbz* exhibit three types of resistance to *S. graminum* biotypes E and I; however, no antixenosis was detected in interactions with biotype K (Zhu et al. 2005). In contrast, plants lacking these genes are more susceptible to damage by insect herbivores (Kumari et al. 2022). This report underscores the importance of plant genetic factors in host resistance.

#### Antibiosis

Antibiosis refers to plant traits that negatively affect the physiology and behavior of insects (Tlak Gajger and Dar 2021). Host plants exhibiting antibiosis can hinder insect growth, increase mortality rates, and reduce reproductive capacity in aphids that feed on them (Gebretsadik et al. 2022a; Palial et al. 2022) (Table 3). For example, antibiosis-resistant wheat genotypes such as Lunxuan 145, Wane, Lunxuan 6, 204,511, Lunxuan 103, and 5215 have been shown to delay aphid growth, increase mortality, and reduce reproduction rates in *S. miscanthi* (Gebretsadik et al. 2022a). Antibiosis resistance is attributed to allelochemical compounds in plants, which may occur naturally or be induced by biotic and/or abiotic stresses (Tlak Gajger and Dar 2021). Certain phenolic compounds in cereal crops negatively impact aphid growth, reproduction, survival, and feeding behavior (Gebretsadik et al. 2022a). For instance, higher levels of 2,4-dihydroxy-7-methoxy-1,4-benzoxazin-3-one (DIMBOA) and its glycoside derivatives in wheat and maize have toxic effects on aphids (Shavit et al. 2018; Silva et al. 2013) (Fig. 4). Additionally, waxy substances on the leaf surfaces of cereal crops (Shavit et al. 2018) and phenolic acids such as ferulic acid, p-coumaric acid, vanillic acid, and caffeic acid in wheat (Gebretsadik et al. 2022a) reduce aphid feeding, population growth, and fecundity, while increasing mortality rates. High levels of hydroxamic acids in wheat plants also inhibit aphid growth, reduce body size, increase mortality, and prolong the pre-reproductive period (Silva et al. 2013).

**Table 3:** Aphid-resistant plant genes and their resistance mechanisms in plants

Source	Resistance genes	Mode of action/ resistance	Reference
Cotton	*GhCAlS5*	Antibiosis and antixenosis to *Aphis gossypii* through callose deposition	(Mbiza et al. 2022)
Cotton	*GhMYC1374*	Antibiosis to *Aphis gossypii* via flavonoids and free gossypol biosynthesis	(Zhang et al. 2023)
Cotton	*GhMYB18*	Tolerance to *Aphis gossypii* via salicylic acid and flavonoid biosynthesis	(Hu et al. 2023)
Cotton	*GhCYP4CJ1*	Antibiosis to *Aphis gossypii* by gossypol and tannic acid biosynthesis	(Ma et al. 2019)
Cotton	*GhLAC4-3*	Mediates lignin-induced antibiosis to *Aphis gossypii*	(An et al. 2023)
Soya bean	*Rag6 loci, Rag3c loci*	Antibiosis resistance to *Aphis glycines*	(Zhang et al. 2017)
Soya bean	*Rag1*, *Rag2*, *Rag3*	Antixenosis to *Aphis glycines*	(Neupane et al. 2019)
Sorghum	*Sgr1*, *Sgr2*, *Sgr7*, *Sgr8*	Antibiosis and antixenosis to *Schizaphis graminum*	(Radchenko et al. 2022)
Wheat	*Gb2*, *Gb3*, *Gb4*, *Gb5*, *Gb6*, *Gb7*, *Gb8*	Resistance to *Schizaphis graminum*	(Luo et al. 2022)
Wheat	*Dn5*	Resistance to *Diuraphis noxia*	(Kisten et al. 2020)
Wheat	*Dn1, Dn2*	Resistance to *Diuraphis noxia*	(Du Toit 1989)
Wheat	*Dn6*	Resistance to *Diuraphis noxia*	(Dong and Quick 1995)
Wheat	*Dn8*	Resistance to *Diuraphis noxia*	(Liu et al. 2001)
Wheat	*Dnx*	Resistance to *Diuraphis noxia*	(Harvey and Martin 1990)

Numerous studies have demonstrated that benzoxazinoids possess antifeedant and toxic properties affecting insect herbivores (Zhou et al. 2018). However, the relationship between benzoxazinoids and aphid resistance remains a topic of debate (Elek et al. 2013; Givovich and Niemeyer 1995; Leszczynski et al. 1992; Zhang et al. 2021). Some researchers have reported a positive correlation between the levels of constitutive benzoxazinoids in host plants and resistance to different aphid species, including *S. avenae*, *Schizaphis graminum*, *Rhopalosiphum padi*, and *Rhopalosiphum maidis* (Thackray et al. 1990; Givovich and Niemeyer 1995; Ahmad et al. 2011). Hydroxamic acids (Hx) have been shown to affect aphids negatively (Makowska et al. 2015; Handrick et al. 2016; Zust and Agrawal 2016). Several studies have established an association between Hx concentrations and wheat's resistance to aphids. In six wheat cultivars, Hx concentrations accounted for a significant portion of the variation in aphid performance (Thackray et al. 1990), and strong correlations have been found between the intrinsic rate of population increase of *S. avenae* and Hx levels (Pereira et al. 2017; Bohdar et al. 1986). Additionally, higher concentrations of DIMBOA in flag leaves, compared to those in the ears at anthesis, were associated with increased *S. avenae* population growth rates in the ears rather than in the flag leaves (Nicol and Wratten 1997). The BRS Timbauva wheat genotype, which had high DIMBOA levels, exhibited strong resistance against *S. avenae* and *R. padi*, while the BRS Guabiju wheat genotype, with the lowest DIMBOA levels, showed reduced resistance to both aphid species (Pereira et al. 2017).

However, other studies suggest that the concentration and composition of benzoxazinoids may not be the primary factors influencing aphid resistance (Pereira et al. 2017; Castañeda et al. 2010; Elek et al. 2013). Aphids may sometimes thrive on plants with higher benzoxazinoid levels (Castañeda et al. 2010). Research has shown no correlation between hydroxamic acid (Hx) levels and wheat resistance to aphids. For example, Hx levels did not correlate with the reproductive response of *Rhopalosiphum padi* across five wheat varieties (Kazemi and van Emden 1992). Similarly, studies on tetra- and hexaploid wheat revealed no correlation between Hx levels and the settlement or reproduction of *R. padi* (Elek et al. 2013). The lack of correlation between Hx levels and plant resistance may be due to low concentrations of these acids, insufficient contact between the aphids and the compounds, the aphid's ability to detoxify or avoid the effects, or the aphid's adaptation to the signals (Pereira et al. 2017). Detoxification enzymes, including P450s, GSTs, and ESTs, exhibited similar activity in aphids feeding on wheat cultivars with varying Hx levels, suggesting that these acids did not induce distinct detoxification responses in *Sitobion avenae* (Castañeda et al. 2009). For instance, GST activity was higher in *S. avenae* fed on moderately resistant wheat cultivars than in those fed on low-resistant cultivars (Leszczynski et al. 1994).

Plants exhibit antibiosis resistance to aphids through oxidative bursts, followed by a hypersensitive response (Goggin and Fischer 2021). This process triggers signaling cascades that activate downstream proteins via phosphorylation, leading to protective responses (Zhang and Zhang 2022). The *Recombination activating gene 6 loci* (*RAG6*) and *RAG3* genes of soybean (*Glycine max*) confer antibiosis resistance to *Aphis glycines* (Zhang et al. 2017). The yelocytomatosis transcription factor (*GhMYC1374*) of cotton plays a key role in this defense against *A. gossypii* by regulating the production of flavonoids and free gossypol (Zhang et al. 2023). Furthermore, the *Callose Synthase* gene family (*GhCAlS5*) (Mbiza et al. 2022), *Cytochrome P450* gene (*GhCYP4CJ1*) (Ma et al. 2019), and *Laccase* gene (*GhLAC4-3*) (An et al. 2023) cotton activates antibiosis resistance against *A. gossypii* in response to initial damage (Table 3). However, most aphid-resistant plant genes do not confer protection against aphids from different species. This is due to variations in aphid feeding behaviors across different plant parts and species, resulting in distinct nutrient intake and responses to the plant's secondary metabolites (Leybourne and Aradottir 2022). For example, the *Dn1* and *Dn5* genes in wheat inhibit the growth of *D. noxia*, but do not reduce the populations of other aphid species (Kisten et al. 2020) (Table 3). Wheat resistance to aphids also varies during different developmental stages, likely due to changes in the secondary metabolic compounds used for plant defense (Dampc et al. 2021; Simon et al. 2021).

#### Antixenosis

Antixenosis is a plant trait that helps prevent insects from locating and settling on their host plants (Painter 1951; Palial et al. 2022) (Table 3). This trait can be influenced by various morphological features, such as trichomes, waxes, pigmentation, and chemical factors, including volatile compounds, enzymes, and secondary metabolites that may repel insects (Stec et al. 2021). Volatile organic compounds, such as *β*-glucosidase and *O*-methyltransferase, exhibit antixenosis effects against *Melanaphis sacchari* (Serba et al. 2021). *O*-methyltransferases produce volatile secondary metabolites derived from phenylpropanoids, flavonoids, and alkaloid biosynthetic pathways (Zhan et al. 2022) (Fig. 4). Ethylene (ET)-mediated pathways contribute to the production of volatile chemicals and the fortification of cell walls during antixenosis responses (Cai et al. 2020). Epicuticular waxes produced on cereal crops reduce the preference for winged aphids and their colonization (Cardona et al. 2023).

Plants release volatiles in response to aphid infestations, which activate lipoxygenase (LOX) activity, triggering the jasmonic acid (JA) signaling pathway and increasing the transcript levels of genes involved in JA-mediated defense (Losvik et al. 2017). Aphid preference varies within and among plant species due to differences in the chemical compounds present in the plants (Loxdale et al. 2020). Volatile organic compounds (VOCs) released by host plants can repel herbivores or attract natural enemies (Loxdale et al. 2020). VOCs released by host plants can repel herbivores or attract natural enemies (Chen et al. 2023). Older wheat plants exhibit a higher level of pre-alighting cues that deter aphid settlement compared to younger plants (Aradottir et al. 2017). Aphids puncture the plant epidermis or mesophyll cells with their stylets, ingesting cytosolic contents (Painter 1951) and decide whether to feed or leave before their stylets reach the phloem (Alvarez and Griffing 2023; Gebretsadik et al. 2022b). Repellent plants experience fewer probes in the epidermis or mesophyll cells than non-repellent cultivars (Stec et al. 2021). The short ingestion period of aphids on phloem sap suggests the presence of a deterrent or aphid-feeding-activated phloem barrier (Gebretsadik et al. 2022b; Stec et al. 2021).

#### Tolerance

Tolerance is a defensive response in plants that enables them to withstand or recover from herbivore damage (Koch et al. 2016; Mitchell et al. 2016) (Fig. 4). Tolerant plants can support high aphid populations without exhibiting damage symptoms such as discoloration or wilting (Enders and Begcy 2021). This process passively restores or synthesizes new photosystem proteins (Peterson et al. 2017). Plant tolerance encompasses compensatory mechanisms that enable the plant to sustain numerous herbivores without adversely affecting the physiology or behavior of the insect pests (Koch et al. 2016; Painter 1951). While the mechanisms underlying plant tolerance to aphids remain largely unknown, they are thought to involve enhanced photosynthetic compensation and ROS scavenging (Shi et al. 2016). Tolerant plants exhibit increased photosynthesis and elevated levels of enzymes that detoxify ROS induced by aphid feeding (Smith et al. 2010; Botha et al. 2006). Research has demonstrated that tolerant plants can enhance their photosynthetic processes by circumventing feedback inhibition and preventing disruptions to electron flow through photosystem II caused by hemipteran insects, including aphid feeding (Cao et al. 2015; Botha et al. 2006; Franzen et al. 2007). Furthermore, the upregulation of peroxidases and other oxidative enzymes during insect feeding, combined with increased phytohormone levels, plays a critical role in promoting plant tolerance to hemipteran insect pests, including aphids (Gulsen et al. 2007; Koch et al. 2016).

Studies have shown that aphid feeding reduces chlorophyll and carotenoid levels in both tolerant and susceptible plants (Liu et al. 2024; Zust and Agrawal 2016). However, tolerant plants can enhance their photosynthetic activities to compensate for this loss (Heng-Moss et al. 2006; Koch et al. 2016; Nietupski et al. 2022). Aphid feeding triggers localized cellular responses, leading to systemic plant reactions (Mertens et al. 2021). The initial injury caused by aphid feeding disrupts sucrose transport, impairs photosynthesis, and affects the transport of essential products (Zust and Agrawal 2016). Prolonged disruption can lead to cell damage from ROS, degradation of chloroplasts, and potentially plant death in susceptible varieties (Zust and Agrawal 2016). In contrast, tolerant cultivars effectively scavenge ROS and detoxify enzymes to protect photosynthesis (Yadav and Chattopadhyay 2023). For example, susceptible wheat lines showed reduced chlorophyll a, b, and carotenoid levels when infested by *D. noxia*, indicating damage to light-harvesting complex II (Heng-Moss et al. 2006). In contrast, resistant wheat lines maintained similar chlorophyll levels despite aphid feeding, suggesting reduced impact from *D. noxia* (Botha et al. 2006). Table 3 illustrates some of the representative plant genes responsible for antibiosis, antixenosis, and tolerance in response to aphid damage.

### Plant innate responses to aphid attacks

Plants interact with various insect pests and develop sophisticated defense mechanisms against insect damage (Hu et al. 2024). These mechanisms primarily involve the production of VOCs that repel aphids, along with numerous physical defense attributes and strategies such as trichomes, cuticles, thorns, and specific leaf colors (Hamann et al. 2021; Gong and Zhang 2014). Additionally, the regulation of plant hormones is crucial for controlling resistance against aphids (Yang et al. 2024; Guo et al. 2023, 2020). However, aphids have evolved mechanisms to circumvent general plant defense strategies against herbivores (Nalam et al. 2019; Zust and Agrawal 2016). Consequently, plants must respond by deploying more specialized defense systems tailored to aphids. Crop plants possess resistance (R) genes and *R* gene homologs that confer resistance to aphids. Specific R gene loci or alleles are associated with effective plant resistance to particular aphids in various crops (Smith 2006), such as the Mi-1.2 gene in tomato, which confers resistance to *Macrosiphum euphorbiae* (Kaloshian 2004), and the virus aphid transmission (Vat) locus in melon, which provides resistance to *A. gossypii* (Dogimont et al. 2014). Notably, these resistance genes belong to the same receptor family (Smith and Clement 2012; Zust and Agrawal 2016), suggesting a shared response to a specific aphid elicitor. Moreover, specific R genes from barley, rye, and wheat provide resistance to *D. noxia* (Smith et al. 1998) and *S. graminum* (Peterson et al. 1998). Research on wheat (Liu et al. 2005) and barley (Nieto-Lopez and Blake 1994) has shown that aphid resistance R gene loci are located on *Triticeae* homoeologous groups 1 and 7.

PRRs also recognize specific molecular patterns of compounds (pathogen-associated molecular patterns, PAMPs) in aphid saliva (Prince et al. 2014; Chaudhary et al. 2014). For example, the activation of PRRs has been linked to increased rates of phloem sealing (Prince et al. 2014). Aphids feeding on resistant peach cultivars exhibit significantly increased probing activity and shorter feeding periods, suggesting enhanced rates of phloem sealing (Sauge et al. 2002). Interestingly, the Mi-1.2-mediated resistance of tomato against the potato aphid is highly dependent on the salicylic acid (SA) pathway (Li et al. 2006; Zust and Agrawal 2016). This suggests the plant may counteract aphid-induced phytohormonal manipulation by linking the SA pathway with an R-gene. Although PRR-mediated and R-gene-mediated recognition are distinct processes, both involve precise mechanisms for the plant to recognize aphid feeding and mount targeted immune responses (Keith and Mitchell-Olds 2013). Although specific receptors or R-genes for detecting aphids have only been identified in a few cases, many plant species possess genotypes or cultivars that are entirely resistant to aphids (Li et al. 2007; Sauge et al. 2006), suggesting the widespread use of *R*-gene-like mechanisms.

Plants have evolved physical and chemical defense strategies against aphids (Singh and Joshi 2020; Mitchell et al. 2016). Plant defense mechanisms against aphids can also be triggered upon herbivore attack (Singh and Joshi 2020; Batyrshina et al. 2020). Plant defense can be categorized as constructive or inductive, depending on the conditions under which the defense mechanism is elicited.

#### Constitutive defense mechanisms

Constitutive defenses are inherent physical and chemical traits that plants have evolved to protect themselves from external threats (Singh et al. 2021). These defenses also include the production of toxins, such as volatile or non-volatile compounds, which inhibit aphid feeding. Constitutive defense mechanisms are present in plant tissues as anticipatory strategies against adverse conditions (Mertens et al. 2021).

Plant physical defenses against aphids include structural modifications, such as cell wall fortification, which create mechanical barriers to feeding (Yang et al. 2024; Nalam et al. 2019). Plant physical features, such as callose, trichomes, and epidermal barriers (e.g., cuticles, waxes, and cell walls), serve as the first line of defense against insects (Singh et al. 2021; War et al. 2012). Furthermore, the role of trichomes in physical defense can be categorized into glandular and non-glandular types (Xie et al. 2011). Glandular trichomes in plants, such as Dendranthema morifolium, store and secrete toxins and mucus as defensive agents to poison or trap aphids. In contrast, non-glandular trichomes are specialized hair-like structures on the epidermis that affect aphid movement and reproductive potential (Xia et al. 2023; Feng et al. 2021). Non-glandular trichomes minimize damage to D. morifolium by hindering aphids from traversing the leaf surface (Dong and Lin 2021). Moreover, the distribution of trichome-based insect resistance can vary across different plant species, tissues, and organs, with younger tissues typically exhibiting more excellent resistance than older ones (Singh et al. 2021).

#### Induced defense mechanisms

Aphid feeding induces changes in the levels of nutrients, phytohormones, and defense proteins in plants as part of the response to aphid-induced damage (Xia et al. 2023). These defense mechanisms aim to repair damaged cells or interfere with aphid nutrient absorption (Appu et al. 2021). This phenomenon is referred to as induced defense. In contrast to constitutive defense, which is continuously active, induced defense is activated only in response to biotic stresses.

### Chemical resistance against aphids

Plant chemical resistance to aphids involves a wide range of defensive compounds and strategies that plants utilize to hinder aphid performance or discourage their presence (Kumaraswamy and Huang 2024). Secondary metabolites, including alkaloids, phenolics, terpenoids, and glucosinolates, are produced by plants to be toxic or repellent to aphids (Divekar et al. 2022). Additionally, plants release volatile organic compounds or non-volatile chemicals that prevent aphids from settling or feeding (Loreto and D'Auria 2022; Divekar et al. 2022).

#### Plant secondary metabolites against aphids

Plant secondary metabolites (PSMs) are crucial in plant–insect interactions, deterring herbivory, hindering digestion, inducing toxicity, and impairing growth and reproduction (Ninkuu et al. 2022; Yang et al. 2024). Additionally, PSMs can attract natural enemies of herbivores (Kundu et al. 2023; Yang et al. 2024). For instance, phenolic compounds negatively impact aphid feeding, growth, and development (Tlak Gajger and Dar 2021). A study on 24 sorghum varieties infested by *M. sacchari* revealed that genotypes with higher polyphenol content exhibited reduced susceptibility to aphid damage (Knoll et al. 2021). In another study, *A. gossypii* infestation enhanced the mRNA accumulation of the *GhMYB18* transcription factor in cotton, which activates the synthesis of SA and flavonoids, thereby boosting cotton's resistance to aphids (Hu et al. 2023). Furthermore, hydroxycinnamic acids (4-caffeoylquinic acid (4-CQA) and 4-p-coumaroylquinic acid (4-pCoQA)) have been identified as key factors in the resistance of apple trees against *Dysaphis plantaginea* (Berrueta et al. 2018). Monoterpenes exert a repellent effect on *M. persicae* by decreasing their average and total probing time on leaves (Dancewicz et al. 2020). Moreover, overexpression of the (*E*)-*β*-caryophyllene synthase gene, terpene synthase 1 (GhTPS1) from *G. hirsutum*, into the R15 cotton variety significantly reduced *A. gossypii* infestation (Zhang et al. 2020).

#### Role of Volatile organic compounds (VOCs) in aphid control

VOCs are natural plant products with low molecular weights and high vapor pressures at room temperature (Effah et al. 2022). These compounds can be classified into several categories based on their biosynthetic origin, including terpenoids, benzenoids, fatty acid derivatives (green leaf volatiles, GLVs), and amino acid derivatives (Aratani et al. 2023; Li et al. 2024; Effah et al. 2022; Ninkuu et al. 2023). VOCs emitted by plants are species-specific, yet they also respond to various biotic and abiotic factors (Effah et al. 2022). Plant VOCs are essential in plant–insect interactions, affecting how insects choose their hosts and serving as signals for feeding, habitat selection, and oviposition for both beneficial insects and herbivores (Effah et al. 2019, 2022). VOCs can function as direct toxins or feeding deterrents and also stimulate secondary plant defense responses when insects feed on plants (Song et al. 2021; Zhou and Jander 2022). Furthermore, VOCs contribute to indirect defense by attracting natural enemies of herbivores, such as predators and parasitoids, to damaged sites (Bezerra et al. 2021).

Plant VOCs also influence the metabolism and behavior of neighboring plants. When plants are under aphid attack, they emit VOCs to trigger aerial defense mechanisms in neighboring plants (Loreto and D'Auria 2022). The signaling pathway involving methyl salicylate (MeSA), SA-binding protein-2 (SABP2), the transcription factors NAC2, and SA-carboxylmethyltransferase-1 (SAMT1) mediates aerial defense responses against aphids and viruses (Gong et al. 2023). The conversion of MeSA to SA activates the NAC2–SAMT1 module, promoting MeSA biosynthesis and triggering immunity against aphids, which in turn reduces virus transmission (Gong et al. 2023). However, certain aphid-transmitted viruses suppress the plant's aerial defense by interacting with NAC2, compromising the plant's immunity to aphid infestation and viral transmission (Gong et al. 2024, 2023).

GLVs are a group of plant-derived compounds consisting of six carbon atoms (C6), including alcohols, aldehydes, and esters, which are produced from fatty acids through the LOX pathway (Ameye et al. 2018). These compounds are emitted by nearly all plants and are triggered by various stressors, such as physical damage, herbivory, fungal or bacterial infections, and environmental stress (Jin et al. 2023; Ameye et al. 2018). This broad-spectrum response to stress suggests that GLVs play a vital role in plants' adaptation to challenging conditions (Jin et al. 2023; Matsui and Engelberth 2022). GLVs are key components of herbivore-induced plant volatiles (HIPVs), where they play crucial roles in plant defense and facilitate chemical communication among plants in nature (Sugimoto et al. 2021). They are involved in various interactions, such as deterring or attracting herbivores and their natural enemies, priming and activating plant defenses, and influencing genes related to stress responses (Matsui and Engelberth 2022; Ameye et al. 2018). GLVs emitted from apple trees infested by Aphis pomi and Dysaphis plantaginea include (*Z*)−3-hexenyl acetate, (*Z*)−3-hexenyl butanoate, (*Z*)−3-hexenyl 2-methyl-butanoate, (*E*)-*β*-caryophyllene, *β*-bourbonene, and (*Z*)−3-hexenyl benzoate (Badra et al. 2021). Additionally, benzaldehyde and (*E*)-*β*-farnesene are specifically linked to *A. pomi*, while linalool and (*E*)−4,8-dimethyl-1,3,7-nonatriene are more closely associated with *D. plantaginea* (Badra et al. 2021). Moreover, exposure to GLVs such as (*E*)−2-hexenal (E2HAL), (*Z*)−3-hexenal (Z3HAL), and (*Z*)−3-hexenol (Z3HOL) induces the accumulation of JA, activates JA-dependent defense genes, and enhances the plant's readiness to respond to future herbivory (Aratani et al. 2023; Tanaka et al. 2023). Engineering plant volatiles for aphid resistance offers an environmentally sustainable alternative for aphid management. VOCs can attract natural enemies and exhibit antifeeding properties, thereby deterring aphids. By manipulating the biosynthetic pathways of VOCs, plants can be engineered to emit higher levels of repellent compounds, such as methyl salicylate or terpenoids. Additionally, enhancing the release of volatiles that attract aphid predators, such as ladybugs or parasitic wasps, can help control aphid populations naturally. This dual approach not only reduces aphid infestation but also promotes a healthier ecosystem by supporting beneficial insect populations.

### Physical Resistance against aphids

Physical resistance in plants to aphids involves structural and morphological traits that form physical barriers or obstacles, effectively deterring or hindering aphid feeding, settlement, and reproduction (Kumaraswamy and Huang 2024; Smith and Chuang 2014).

#### Plant cell wall biosynthesis confers physical resistance to aphids

The plant cell wall plays a critical role in protecting against aphid infestation by providing both physical and chemical resistance (Xia et al. 2023). It prevents the penetration of aphid stylets into plant tissues and disrupts their ability to feed on phloem sap (Twayana et al. 2022). These protective traits are largely attributed to the cell wall's nearly impermeable nature, conferred by a variety of phenolic compounds, including lignin and pectin (Hu et al. 2018; Liu et al. 2018).

Resistance to aphids in plants is primarily mediated by two mechanisms that involve alterations in the structure of the plant cell wall (Xia et al. 2023). The first mechanism is based on the activity of critical genes that modify the cell wall’s composition and structure, enhancing its resistance to aphids. The second mechanism, on the other hand, involves the hydrolysis of cell wall polysaccharides by aphid saliva, which in turn triggers a defensive response in plant tissues (Leybourne and Aradottir 2022; Xia et al. 2023). These two mechanisms often act synergistically.

#### Pectin

Pectin is a critical component of plant cell walls and plays a key role in modulating cell wall resistance to aphids (Xia et al. 2023). It consists of three main polysaccharide domains: homogalacturonan (HG), rhamnogalacturonan I (RG-I), and rhamnogalacturonan II (RG-II) (Stratilova et al. 2020). Aphid saliva contains several cell wall-modifying enzymes (CWMEs), including pectin methylesterases (PMEs), polygalacturonases (PGs), and pectate lyase enzymes (PLs), which break down cell wall polysaccharides to facilitate nutrient uptake (Silva-Sanzana et al. 2020). During this process, HG is de-methyl-esterified by PMEs, producing oligogalacturonides (OGs) (Silva-Sanzana et al. 2020). Plants recognize OGs as damage signals and initiate a defense response via the mitogen-activated protein kinase (MAPK) signaling pathway (Hou et al. 2019; Jiang et al. 2022). The demethylesterification of HG is critical for triggering an effective plant defense response against aphids (Osorio et al. 2008). Infestation by the aphid *Myzus persicae* rapidly increases pectin-PME activity in Arabidopsis leaves, leading to higher levels of demethylated pectin and the release of methanol (Silva-Sanzana et al. 2019). This process elicits a defense response, as the increased methanol emissions act as volatile signals recognized by both plants and insects (Dorokhov et al. 2012). For example, tobacco plants overexpressing PME, which exhibit higher methanol emissions compared to the wild-type genotype, showed a dramatic increase in resistance to various phytophagous insects, including aphids and whiteflies (Dixit et al. 2013; Silva-Sanzana et al. 2019).

#### Lignin

Lignin is another key component of the cell wall that enhances mechanical support and provides both physical and chemical barriers to biotic and abiotic stresses (Kaur et al. 2022; Ninkuu et al. 2022). It plays a crucial role in a plant's resistance to insect pests and pathogens (An et al. 2023). In response to internal and external signals, plants regulate the timing, location, amount, and type of lignin deposition (Reyt et al. 2021). Increased lignin deposition helps protect cells from insect puncturing, reduces the susceptibility of cell walls to cell wall-degrading enzymes (CWDEs), and limits the spread of toxins released by insects (Hu et al. 2018; Yadav and Chattopadhyay 2023). Elevated lignin content in the epidermis impedes aphid access to vascular tissues, thereby providing resistance to aphid infestation (Cardona et al. 2022; Yang et al. 2024). The transcription factors MYB30, MYB55, and MYB110 play key roles in plant immunity by upregulating genes involved in the monolignol biosynthesis pathway in rice (Kishi-Kaboshi et al. 2018). Overexpression of *CmMYB15* and *CmMYB19* in chrysanthemums enhanced resistance to the aphid *Macrosiphoniella sanborni* by regulating lignin biosynthesis genes, leading to increased lignin deposition and cell wall thickening (An et al. 2019; Wang et al. 2017). This MYB transcription factor activates genes involved in lignin production, providing physical defense against aphids. Silencing the expression of G*ossypium hirsutum non-specific lipid transfer proteins* (*GhnsLTPsA10*) in cotton increased lignin content, enhancing aphid resistance (Chen et al. 2021). Similarly, treatment with methyl jasmonate and endophyte infection significantly increased lignin concentration, reducing aphid populations (Zhao et al. 2022). Lignin also reduces insect nutrient availability by hydrogen bonding with proteins and carbohydrates, potentially acting as a toxin (Liu et al. 2023; Yadav and Chattopadhyay 2023).

#### Callose deposition

Callose is a linear polysaccharide found in plant cell walls, primarily composed of β−1,3-linked glucose residues with occasional β−1,6-linked branches (Li et al. 2023). It is predominantly produced during cell wall formation and plays a critical role in the structure of plant vascular bundles, microspore outer walls, and dividing cell plates (Li et al. 2023). Callose rapidly accumulates in cell plates, microspores, sieve plates, and plasmodesmata in response to insect damage and other stress factors (Li et al. 2023). It functions as a primary defense mechanism against biotic and abiotic stresses (Nawaz et al. 2023). Overexpression of *GhCalS5* in cotton enhanced resistance to *A. gossypii* by promoting increased callose deposition in cotton leaves (Mbiza et al. 2022). This accumulation blocks the flow of phloem sap, preventing aphids from efficiently accessing nutrients and thereby slowing their reproduction rate (Yang et al.^2024^). Various signaling pathways and biological processes, including hormonal signaling regulate callose synthase activity. For instance, ABA enhances callose synthase activity in rice, promoting callose deposition as a defense against *Nilaparvata lugens* (Liu et al. 2017). Given the critical role of plant cell walls in aphid defense, breeders could target key regulators of cell wall biosynthesis to develop cultivars with enhanced resistance to aphid penetration. Additionally, incorporating genes that produce anti-aphid proteins or secondary metabolites into the cell wall could provide biochemical solutions to mitigate aphid infestation.

### Plant hormones against aphids

Plant phytohormones such as JA, SA, ET, abscisic acid (ABA), and gibberellic acid (GA) play crucial roles in regulating plant defense mechanisms against aphid infestations (Gao et al. 2007; Morkunas et al. 2011). In mustard plants, activation of JA-mediated defense responses has been shown to suppress the growth of Lipaphis erysimi populations (Yang et al. 2024). Furthermore, JA produces defense-related enzymes and compounds, which help plants resist insect attacks. Soybean plants with increased aphid tolerance exhibit elevated JA levels (Chapman et al. 2018). Recent studies have also highlighted that oxo-phytodienoic acid (OPDA), an important intermediate in the JA biosynthesis pathway, promotes callose accumulation, thereby enhancing resistance to corn aphids (Varsani et al. 2019). Aphid-induced SA signaling varies depending on the plant and aphid species involved. For instance, in sorghum, wheat aphid feeding significantly upregulates genes associated with SA-mediated defense responses more than infestations by *Melanaphis sacchari* (Yang et al. 2024). Additionally, the R2R3 MYB transcription factor GhMYB18 enhances cotton plant resistance to aphids by regulating SA and flavonoid biosynthesis (Hu et al. 2023). The application of ABA and GA to uninfected barley leaves did not replicate the rolling or streaking damage symptoms caused by the aphid D. noxia (Miller et al. 1994). ABA plays a crucial role in the insect defense response of several plant species following aphid infestation. For instance, in sorghum, several ABA-upregulated genes enhance cell wall strength, thereby improving resistance to S. graminum (Park et al. 2006). Aphid infestation increases ethylene (ET) production in the aphid-tolerant Frontera cultivar compared to the susceptible Aramir cultivar (Morkunas et al. 2011).

### Aphid-resistant crop breeding: Historical perspectives and development trends

Sustainable crop production is critical for ensuring global food security, particularly in the face of environmental uncertainties (Benitez-Alfonso et al. 2023). Aphids represent a significant threat to crop production, causing yield losses that undermine food security. These pests feed on plants and transmit harmful viruses, leading to substantial reductions in the yield of major field crops (Singh et al. 2022). Host plant resistance (HPR) refers to a plant's ability to resist colonization and feeding by insect herbivores (Koch et al. 2016; Enders and Begcy 2021). It is considered the most efficient and sustainable strategy for managing aphid infestations, including those caused by *D. noxia* (Ward et al. 2020). The development of pest-resistant plants involves understanding the genetic basis of resistance and identifying appropriate sources for breeding programs (Lefebvre et al. 2020). Efforts to breed wheat varieties resistant to *D. noxia* began in South Africa in the late 1980s, leading to the identification of the *Dn1* and *Dn2* resistance genes (Du Toit 1989). To date, nineteen genes conferring resistance to D. noxia have been reported, most of which map to specific chromosomal regions (Ward et al. 2020). For example, *Dn1*, *Dn2*, *Dn5* (Du Toit 1989), *Dn6* (Saidi and Quick 1996), *Dn8* (Liu et al. 2001), and *Dnx* (Harvey and Martin 1990) have been mapped to the 7D chromosome of wheat Most of these genes have been identified in wheat accessions from diverse geographic regions. As of now, forty-three cultivars exhibiting resistance to *D. noxia* have been released (Kisten et al. 2020). However, various biotypes of *D. noxia* have overcome multiple host plant resistances globally, demonstrating the pest's ability to circumvent genetic resistance mechanisms developed through plant breeding (Puterka et al. 1992).

Recent advancements in molecular plant breeding have facilitated the development of accessible and precise methods for studying resistance traits. Techniques such as quantitative trait loci (QTL) mapping, genome-wide association studies (GWAS), and bulked segregant analysis (BSA) are essential tools for uncovering the genetic basis of traits like aphid resistance. Researchers have utilized a range of molecular markers, including amplified fragment length polymorphism (AFLP), simple sequence repeats (SSR), and single nucleotide polymorphisms (SNP), to construct genetic linkage maps and identify QTLs associated with aphid resistance. These analyses have been applied to various crops, including wheat, maize, soybean, and cucumber (Zhang et al. 2017; Singh et al. 2023). For instance, in cotton, QTL mapping using an F2 population derived from the aphid-resistant cultivar Xinluzao 61 and the aphid-sensitive cultivar Xinluzao 50 identified the *GhLAC4-3* gene, which encodes an enzyme involved in lignin synthesis, as a significant contributor to aphid resistance (An et al. 2023). In pepper, QTL mapping identified the Rmpas-1 and Rmprp-1 loci, which are associated with reduced aphid survival and reproduction (Sun et al. 2020). The combined use of SSR and SNP markers has proven effective in mapping a novel *Aphis craccivora* resistance locus in the cowpea breeding line SARC 1–57-2 (Kusi et al. 2018). This approach has also played a crucial role in introducing resistance traits into elite cultivars through marker-assisted backcrossing.

Emerging genomic techniques such as GWAS and BSA have greatly enhanced the identification of aphid-resistance genes. GWAS allows for the detection of sequence variants, specifically SNPs, across the plant genome, enabling the identification of SNPs associated with aphid-resistant traits (Dehghan 2018). A GWAS conducted on Pisum sativum resistance to both a pea-adapted biotype and a non-adapted biotype of *Acyrthosiphon pisum* revealed a major genomic region on chromosome 7 that confers resistance to both biotypes. This region, designated as the major-effect QTL *ApRVII*, is associated with strong linkage disequilibrium blocks that correlate with resistance to one or both aphid biotypes (Ollivier et al. 2022). BSA has also proven effective in identifying aphid resistance traits by selecting parent individuals exhibiting distinct phenotypic extremes for breeding. In peaches, mapping identified the *Rm3* locus on chromosome 1, which is localized to a 160-kilobase region containing 21 genes (Pan et al. 2022).

## Conclusion and future perspectives

Aphids pose a significant agricultural risk through nutrient extraction and disease transmission to host plants (Skendzic et al. 2021). The economic impact of aphids is influenced by factors such as population size, infestation timing, and feeding duration (Radchenko et al. 2022). Understanding aphid population dynamics is essential for predicting abundance and developing effective management strategies. Abiotic factors, including climate and topography, influence aphid populations, prompting migration to more favorable environments. Climate change further complicates predictions of aphid abundance by affecting their distribution, survival, and interactions with plants and other species (Li et al. 2020).

This review explores methods for controlling aphids, including EPF and host plant defenses like antibiosis, antixenosis, and tolerance. It also discusses the role of plant cell walls and GLVs in response to aphid feeding. The development of EPF shows promise as an eco-friendly aphid control method due to its natural ability to infect aphids and its safety for non-target organisms (Kinley et al. 2021).

Plants serve as hotspots for sap-sucking insects such as aphids, which extract nutrients and energy from their host plants to grow and reproduce. In response to these threats, plants have evolved a range of defense mechanisms aimed at protecting themselves from aphids, which feed on the nutrient-rich phloem sap. These defenses involve resource reallocation, along with the production of defensive metabolites and physical structures. Plant trichomes and the cell wall serve as protective barriers and trigger defensive responses to aphid infestations. Lignin, a key component of the cell wall, provides physical protection against aphids. Increased lignin deposition helps safeguard cells from insect piercing, reduces the cell wall's vulnerability to degrading enzymes, and restricts the spread of insect-released toxins. PSMs play a crucial role, functioning as toxins, feeding deterrents, or signaling the activation of defense responses. Plant hormones, such as JA, SA, ET, ABA, and GA, also regulate defense pathways against aphids. Additionally, plants employ phloem-sealing mechanisms, including sieve tube blockages, callose deposition, and ROS production, as essential resistance traits to combat aphid attacks (Nalam et al. 2019). Callose rapidly accumulates in response to aphid feeding, forming barriers in cell plates, microspores, sieve plates, and plasmodesmata. Overexpression of the GhCalS5 gene enhances cotton resistance to aphids by increasing callose formation, blocking phloem sap flow, and slowing aphid reproduction.

However, aphids can evade these defenses using salivary effectors that manipulate plant hormones (Guo et al. 2020). In response, plants convert aphid saliva components into signals that activate defense mechanisms, including R genes and PRRs that detect salivary compounds and trigger immune responses.

Recent advancements in molecular breeding have improved crop yields and increased resistance to various stresses. The focus is now on developing superior crop varieties with optimal genotypic combinations that provide broad-spectrum resistance to insect pests. Innovations in plant genetics and biotechnology offer opportunities for sustainable pest management. Stacking multiple resistance genes has enhanced crop durability and reduced the likelihood of pests developing resistance. Strategies like refuge crops and high toxin expression can help delay resistance to transgenic crops. Modern genomic breeding technologies, including genomic selection and haplotype-based breeding, QTL, GWAS, and BSA, facilitate the creation of aphid-resistant crops through better combinations of resistance alleles. While genetically modified (GM) crops are becoming more common, food safety and resistance management remain important. Before implementation, evaluation of local agricultural systems and pest biology is essential for ensuring the long-term effectiveness of resistant crops.

Integrated Pest Management (IPM) is a cornerstone of aphid control strategies, emphasizing the integration of biological, cultural, physical, and chemical methods for sustainable pest management. Effective implementation requires advanced monitoring systems, including remote sensing and AI-driven data analysis, to track aphid populations accurately. While natural predators like ladybugs and lacewings can help manage aphids, their effectiveness in commercial plantations can be limited by their tendency to switch prey. Therefore, incorporating advanced technologies into IPM practices can enhance aphid control. To effectively combat aphids and other pests, collaborative research involving experts in genetics, insect physiology, AI, agronomy, biochemistry, and climatology is vital for developing integrated pest management solutions.

## Data Availability

Data sharing does not apply to this article as no datasets were generated or analyzed during the current study.

## Abbreviations

ABA: Abscisic acid
AFLP: Amplified fragment length polymorphism
AI: Artificial intelligence
BSA: Bulked segregant analysis
CWMEs: Cell wall-modifying enzymes
EPFs: Entomopathogenic fungi
ET: Ethylene
GA: Gibberellic acid
GM: Genetically modified
GLVs: Green leaf volatiles
GWAS: Genome-wide association studies
HG: Homogalacturonan
HIPVs: Herbivore-infested plant volatiles
IPM: Integrated Pest Management
ISR: Inducing systemic resistance
JA: Jasmonic acid
LOX: Lipoxygenase
MAPK: Mitogen-activated protein kinase
MeSA: Methyl-salicylate
OPDA: Oxo-phytodienoic acid
PAMP: Pathogen-associated molecular patterns
PGs: Polygalacturonases
PLs: Pectate lyase enzymes
PMEs: Pectin methylesterases
PRRs: Plant pattern recognition receptors
PSMs: Plant secondary metabolites
R-gene: Resistance genes
QTL: Quantitative trait loci
ROS: Reactive oxygen species
SA: Salicylic acid
SABP2: Salicylic acid-binding protein -2
SAMT1: Salicylic acid-carboxylmethyltransferase-1
SNP: Single nucleotide polymorphism
Vat: Virus aphid transmission
SSR: Simple sequence repeat
VOCs: Volatile organic compounds
